# Dr Eye

**DOI:** 10.1186/2043-9113-5-S1-S21

**Published:** 2015-05-22

**Authors:** Ioannis Karatzanis, Kostas Marias, Vangelis Sakkalis

**Affiliations:** 1 FORTH, Νikolaou Plastira 100, Vassilika Vouton, GR-711 10, Heraklion, Crete, Greece

## Characterisation

Tool (Native Windows Application), imaging, DICOM. Upon request the tool may be provided for trial use or under a license agreement.

## Tool description

**Figure 1:**
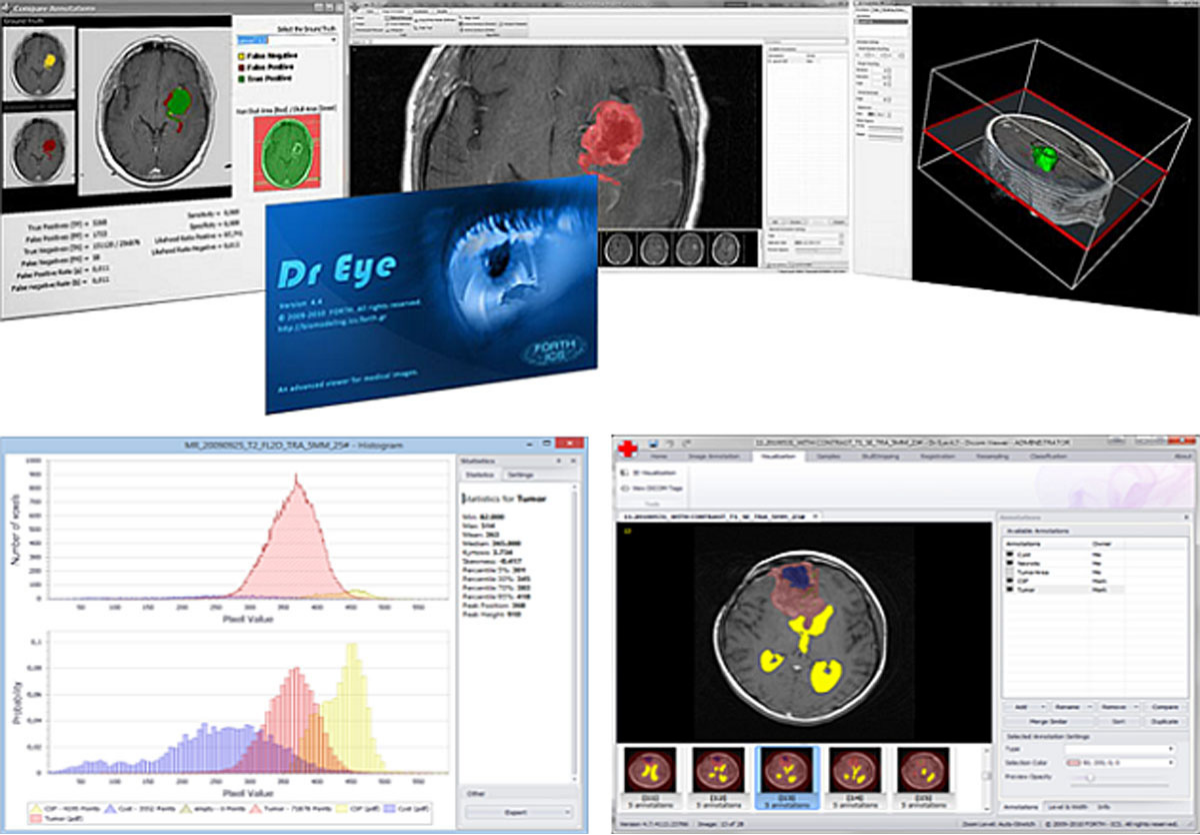
Dr Eye’s user interface. From top left to bottom right: comparison module with statistics among two different annotations in the same slice; main workspace with one annotation colored in red; 3D visualization of the series; multiple annotations selected with different coloring; histograms and statistics for each of the annotations.

Dr Eye viewer is an open access, flexible and easy to use platform for the intuitive annotation and segmentation of tumor region images (Figure 1). Its clinically driven development followed an open modular architecture focusing on plug-in components [1]. Dr Eye’s main advantage is that the user can quickly and accurately delineate complex areas in medical images. Additionally, multiple labels can be set to allow the user to annotate and manage many different areas of interest in each selected slide. The close collaboration with clinicians in designing the platform has ensured that it can be effectively used in the clinical setting.

This tool is suitable for use by various clinicians such as radiologists or oncologists, and by anyone who needs to view DICOM images, or perform measurements and analysis of their imaging data (Figure 1). It can also be part of the workflow of any clinical environment, from health centers to hospitals and it supports usage as a tool in clinical trials or even as training tool in an educational environment. Dr Eye’s main purpose is to enable clinicians to efficiently and intuitively annotate large numbers of 3D tomographic datasets. Both manual and well-known semi-automatic segmentation techniques are available allowing clinician to annotate multiple regions of interest during the same session. Additionally, it includes features like contour drawing, refinement and labeling that assist in the delineation of tumors. Segmented tumor regions can be annotated, labeled, deleted, added and redefined. Interaction with PACS systems is possible with DICOM Query / Retrieve.

The platform has been tested with hundreds of MRI datasets to assess and improve usability, extensibility and robustness. Technically it is based on the .NET framework architecture and can be used in any Windows based computer.

## Status of development

Version 5.7 released on 1.5.2014, beta version.

## Users

Oncologists, radiologists and clinicians with interest in handling DICOM images.

## Links

http://biomodeling.ics.forth.gr and http://biomodeling.ics.forth.gr/?page_id=8
